# Evaluation of a Porcine deltacoronavirus eradication program in a full-cycle pig farm in Peru

**DOI:** 10.5455/javar.2021.h515

**Published:** 2021-06-23

**Authors:** Jhonas Vicente-Huaman, Oscar E. Gómez-Quispe

**Affiliations:** Faculty of Veterinary Medicine and Animal Science, Universidad Nacional Micaela Bastidas de Apurímac, Abancay, Perú

**Keywords:** Coronavirus, diarrhea, feedback, piglets, RT-PCR, viral exposure

## Abstract

**Objective:**

In this case report, we report for first the time the presence of porcine deltacoronavirus (PDCoV) in Peru (October 2019) and Latin America, and we present a control and eradication program using feedback (exposure)/controlled homogenization in a pig farm.

**Materials and Methods:**

This farm is located in the eastern jungle of Peru. Initially, clinical signs that appeared to be infectious diarrhea were detected, but the disease rapidly progressed to green diarrhea, vomiting, and increased mortality in piglets. These symptoms were compatible with those produced by porcine epidemic diarrhea virus and transmissible gastroenteritis virus, but also with PDCoV. Because the disease could not be diagnosed by clinical signs and symptoms, analysis by real-time polymerase chain reaction was used. Implementation of a feedback/controlled homogenization program was quickly planned, accompanied by the closure of the farm, animal and farm health strategies, and its respective monitoring.

**Results:**

At the farm level, between 1 and 9 weeks after application of the program, the samples were positive for PDCoV, but at week 10, they were negative. At week 12, the weaned and fattening piglets gradually became populated as negative animals. In the follow-up before the opening of the farm, all the piglets were negative. In the final verification, gilts (week 35) entered the breeding area as replacements only after being tested negative for PDCoV.

**Conclusion:**

A rigorous feedback/controlled homogenization program and complementary measures led to eradicating PDCoV from the farm.

## Introduction

Coronaviruses are viral agents responsible for respiratory and gastroenteric diseases in pigs. These comprise three genera, Alphacoronavirus, Betacoronavirus, and Gammacoronavirus, to which Deltacoronavirus has been recently added [1,2].

Since the Deltacoronavirus was first identified in Hong Kong in 2012, the virus has crossed borders, reaching North America in 2014 [3], Korea, China, and Thailand in 2016 [4,5]. The virus causes severe diarrhea and mortality between 30% and 40% in piglets less than 21 days old [6], and produces a highly epidemic and endemic disease [7]. Until 2019, this disease had not yet been reported in Colombia and Peru [8] and other countries in Latin America.

The clinical manifestations and clinical signs of this disease, such as acute enteritis, watery diarrhea, vomiting, and extreme dehydration up to 15% [6], can be confused with those produced by the porcine epidemic diarrhea virus (PEDV) and the transmissible gastroenteritis virus (TGEV) [7]. Regarding the organization of the porcine deltacoronavirus (PDCoV) genome, starting from 5′-end, it has a 5′ untranslated region (UTR), replicase (ORF 1a/b), spike (S), envelope (E), membrane (M), non-structural 6 (NS6), nucleocapsid (N), NS7 genes and 3′-UTR [1,9], and lacks ORF3 and NS1, as in others coronaviruses [10]. This PDCoV not only causes serious diarrhea in suckling piglets but also possesses the potential for cross-species transmission [11].

In this context, it has been recommended to have a simple, rapid, and highly sensitive diagnostic method for its detection [7], because it is urgent to prevent, control, and avoid the spread of the virus to the environment. The safest method to detect PDCoV is through real-time polymerase chain reaction (RT-PCR) [9] or quantitative real-time reverse transcription (qRT-PCR) [12]. Currently, there are no effective treatments and vaccines against PDCoV infection [13]. Regarding the control and eradication of the disease in farms with similar pathogenesis, feedback or exposure [14] and controlled homogenization have been used.

Because this disease produces great economic losses by the mortality of suckling piglets [6], in this report, we provide information on the presentation, diagnosis, control, and eradication of this disease in a pig farm of Peru.

### Clinical history

**Figure 1.**
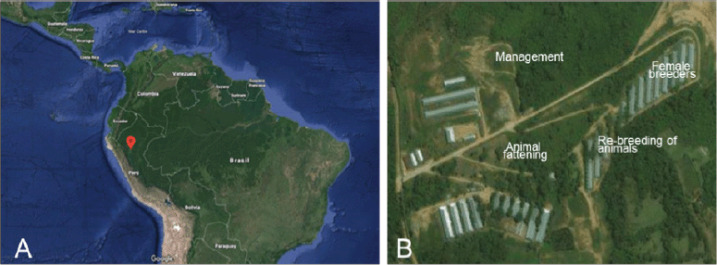
(A) The location map of the area where the clinical case was presented and (B) the distribution of the production areas of the pig farm.

The clinical case occurred in a full-cycle monosite farm located in the eastern jungle (department of San Martin) of Peru (Fig. 1A), where there were 1,350 mothers in production. According to the data on the health status of the pig farm, it was free of enteric viral diseases, such as PEDV, porcine gastroenteritis virus (GETV), and PDCoV. The pig farm is located in an area far from the urban area. It had a medium biosecurity system, which included perimeter fencing, washing and disinfection area for vehicles, dry showers and showers with water, and restricted internal traffic areas (Fig. 1B), according to official protocols (R001.2018.DESMA).

**Figure 2.**
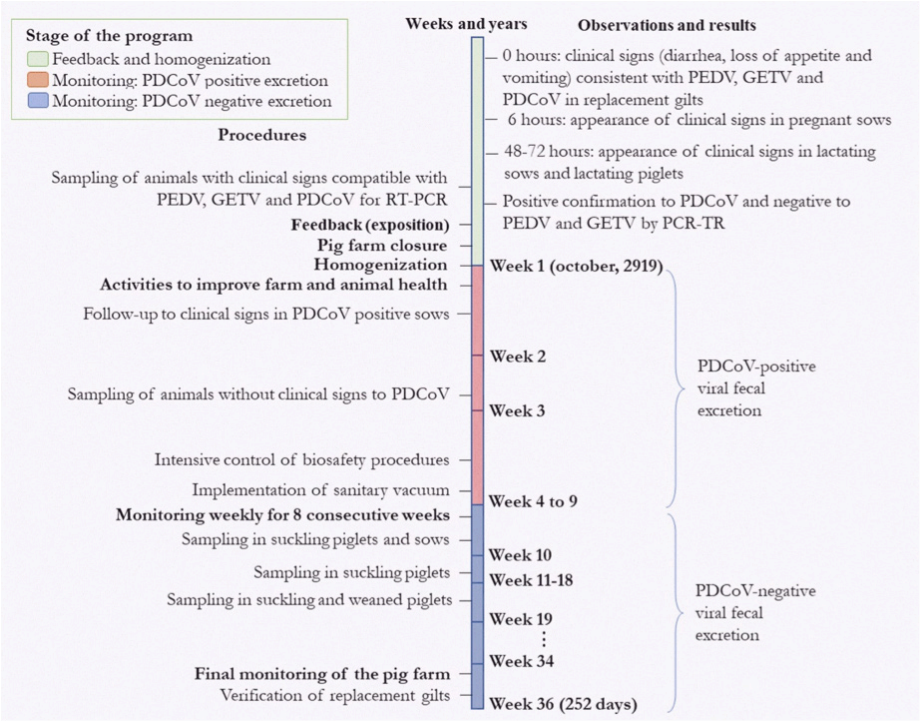
Timeline of a feedback (exposure) and homogenization program for the eradication of PDCoV in a pig farm.

At the end of October 2019 (week 1), as shown in the timeline (Fig. 2), in this farm, inappetence, green watery diarrhea with a fetid odor, and vomiting were observed in the replacement piglets from 70 to 170 days old. Severe dehydration was not observed, and body temperature was within normal parameters. The clinical signs observed made us suspect a bacterial disease, such as *Salmonella*, *Lawsonia*, or *Brachyspira*, but they were also compatible with those produced by PEDV or GETV. Six hours later, clinical signs were observed in 30% of pregnant sows and 8 h later in mothers and suckling piglets of different ages. At 48 h after the presentation of the first cases, the loss of appetite was 90%. Between 48 and 72 h, the percentage of diarrhea increased to 90% in suckling piglets and pregnant sows to a prevalence of 30%.

### Disease diagnosis

Due to the clinical evidence and the rapid spread of the disease, it was not possible to diagnose the causative agent of the disease. Two days after the first infectious cases were observed, a differential diagnosis was requested to PEDV, TGEV, and the possible PDCoV by molecular techniques.

Samples of animals with clinical signs compatible with the previously mentioned diseases were sent to two laboratories. The first shipment of samples was made to the Private Laboratory LASSER-Jallavet SAC (Lima, Peru), which consisted of three samples: intestinal content of lactating piglets, diarrhea of pregnant sows, and diarrhea of lactating sows. The result by qRT-PCR was Ct 09 (cycle threshold value), which indicated that the samples had a high viral load [15] for PDCoV, but negative for PEDV and GETV. In order to confirm the results of the previous analysis, the second shipment of three other samples, diarrhea from pregnant sows and intestinal contents of lactating piglets, was made to the Laboratory of Microbiology and Parasitology of the Faculty of Veterinary Medicine of the Universidad Nacional Mayor de San Marcos. The result by RT-PCR was positive for PDCoV, with Ct 13.39 in pregnant sows and Ct 09 in suckling piglets, while the tests were negative for PEDV and GETV [16].

Both laboratories had confirmed by qRT-PCR and RT-PCR that the samples were positive for PDCoV, and with these results, the presence of PDCoV was evidenced in Latin America and Peru in 2019.

### Control and eradication program

Once the agent was diagnosed, a program to control and eradicate PDCoV disease was quickly designed and implemented, which had four components, viral exposure (feedback) and controlled homogenization, closure of the farm, complementary activities to improve the animal health and pig farm, and monitoring [8,14], according to protocols and authorizations of agricultural health authority (National Service of Animal Health in Peru) of the country (R0016.2019. MINAGRI-SENASA-DESMA).

The application of feedback [8,14] to PDCoV was carried out 92 h (week 1) after presenting the first clinical cases. The purpose was to induce immunity in the pig farm. This controlled dissemination technique consisted of dosing orally with 10 ml of infective material to all breeding sows, semen donors, and replacement gilts. Infectious material used was the shredded intestinal content of piglets [17] with severe clinical signs of the disease [6], in non-chlorinated water. Homogenization [8,14] consisted of infecting all the animals that still showed clinical signs compatible with PDCoV.

These activities were accompanied by the closure of the pig farm to all entries and exits of animals, people, and vehicles for 4 months. In addition, complementary activities to improve animal health and pig farm were carried out, such as (a) the identification and registration of sows with clinical signs compatible with PDCoV (24–72 h post-feedback) in order to apply homogenization, (b) support for piglets in the consumption of colostrum, which included washing and disinfecting the body and udders of their mothers, (c) rigorous washing and disinfection of environments, (d) application of sanitary vacuum, and (e) strong restriction of the transit of workers in production environments (reproduction, rearing, and fattening).

Two methods were used to disinfect the farm. Chemical disinfection for equipment and surfaces in which calcium hydroxide (1 kg of lime/l of water) and 45.3% potassium monopersulfate (10 g/l of water) were used, as well as a commercial product virocid^® ^(Bayer, Spain) where every 100 ml contained 7.0 g of alkyldimethylbenzylammonium chloride, 14. 6 g of isopropanol, 10.7 g of glutaraldehyde, 7.8 g of didecyldimethylammonium, and 2 g of pine oil at a dose of 5 ml/l of water. In the physical disinfection, flamethrowers were used.

Monitoring [8,14] was carried out in three phases. The first consisted of the daily search by observing animals with clinical signs compatible with PDCoV (diarrhea, vomiting, and loss of appetite), their identification, and registration. The purpose was to know the progress of the effect of the feedback and homogenization exposure technique. Second, 7 days after feedback and homogenization, 15 excreta samples from 14% of sows that did not present any of the clinical signs were sent to the laboratory to verify virus presence by qRT-PCR. The samples sent belonged to animals that did not have clinical signs, despite having been inoculated with infective material during the feedback. These samples were taken using a rectal swab and impregnated on FTA cards^®^ (Flinders Technology Associates, UK). Third, after 60 days post-feedback and controlled homogenization, samples were sent to the laboratory every week to determine negativity in suckling piglets. The activity was carried out until completing four consecutive negative results in 100% of the samples [17,18], which would lead to declaring the reproduction area free of viral excretion. Similarly, we proceeded with the animals in other productive environments.

## Results

### Weight of suckling piglets and mortality

**Figure 3.**
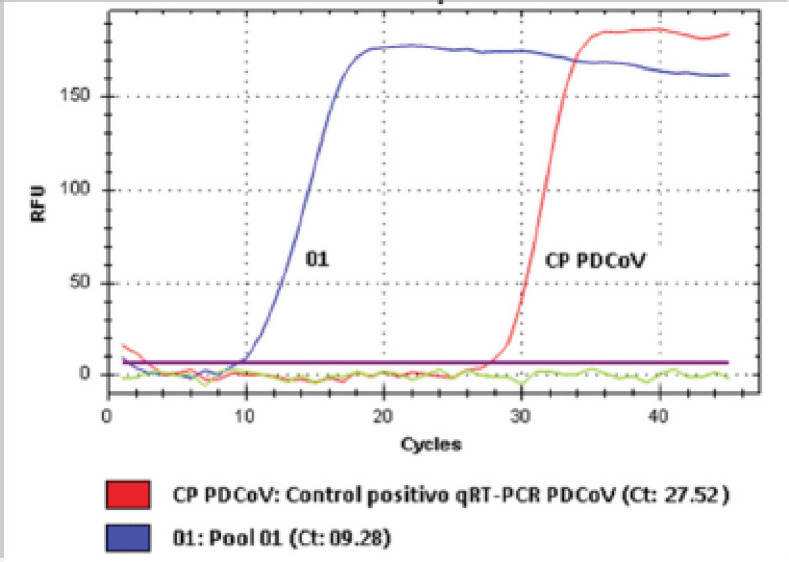
qRT-PCR analysis shows positive control and presence of PDCoV.

Before PDCoV disease, weaning weight and mortality of suckling piglets, shown in Table 1, were 6.45 kg and 3.66%, respectively. In the same table, during the disease, pre-weaning mortality increased up to 24%, producing a decrease in weaned piglets, and in addition, the body weight decreased, reaching an average of 4.94 kg. Post-feedback and homogenization, mortality and weaning weight both returned to the initial values in the indicated table. While the feedback and homogenization were being implemented, the piglets were rehydrated with sodium chloride physiological solution (0.9% NaCl) and acidifiers (Acid pack 4 way®; Altech, USA) orally. Despite this, the mortality of piglets (1–14 days old) due to dehydration, caused by diarrhea in the first hours of the disease, could not be controlled. This persisted for 4 consecutive weeks, causing a decrease in weaning weight and a reduction in the number of piglets weaned (Table 1). After implementing or post-feedback and homogenization, 80% of the inoculated sows presented at least one of the compatible clinical signs (diarrhea, vomiting, or loss of appetite). Of the 20% of the remaining sows, which were inoculated but did not show any visible and compatible clinical signs, 15 samples were randomly taken from the same number of individuals to verify the presence of PDCoV. According to the qRT-PCR results (Fig. 3), all 15 (100%) samples were positive for PDCoV (Table 2), which indicated that the feedback and homogenization had worked.

**Table 1.** Mortality of suckling piglets and weaning weight before the disease, during and after feedback and homogenization to PDCoV.

Weeks		Born alive		Suckling piglet mortality			Weaning weight^a^ (kg)
				*n*		%	
Before the disease						3.66	6.45
	4		760		29	3.82	6.60
	3		768		28	3.65	6.52
	2		755		20	2.65	6.40
	1		728		33	4.53	6.26
During PDCoV disease						20.58	4.94
	1		750		135	18.00	5.17
	2		722		124	17.17	4.23
	3		763		177	23.20	4.64
	4		748		179	23.93	5.71
After feedback and homogenization						2.33	6.03
	5		742		24	3.23	6.01
	6		749		14	1.87	6.06
	7		716		15	2.09	5.97
	8		756		16	2.12	6.07

a Average weaning weight.

### Post-feedback monitoring and homogenization

10At week 10 (Fig. 2), 40 samples were taken from suckling piglets of the second and third week of age (five samples in each pool) with low weights and suspected of diarrhea. Additionally, 20 sow excreta samples were taken. The analysis reports showed that all the samples were negative for PEDV and PDCoV. These first results showed that the program had satisfactory results. On the farm, the procedures of the implemented program were made even more rigorous to eradicate PDCoV disease from the production area (breeding boar and breeding sows).

### Weekly monitoring of piglets

After the first satisfactory result of the feedback and homo-genization program, the excreta of suckling piglets began to be monitored. For this purpose, 32 samples were sent per week to the laboratory. The results showed that 100% of the samples were negative for the presence of PDCoV; so, according to the laboratory, the farm was already free of the presence of PDCoV. With these results, starting at week 12, the rearing areas for weaned and fattening piglets were gradually populated with negative animals.

### Final monitoring prior to the opening of the farm

At the end of the program, stool samples were taken from the breeding and rearing areas sent to the laboratory for analysis. The results gave a PDCoV negative animal condition (data not shown). Although monitoring continued in each of the production phases, PDCoV disease had already been eradicated from the pig farm with the consequence of having obtained large economic losses.

### Verification in gilt sows

At week 35, as shown in Figure 2, 295 gilt sows entered the reproduction area as replacements in order to improve the productive parameters of the breeding stock. However, this was only possible when these animals received the negative condition to the PDCoV virus. According to the RT-PCR analysis report, all were negative for PDCoV (Table 3).

## Discussion

The clinical symptoms of PDCoV are similar to those produced by PEDV and TGEV in piglets [19], and PDCoV disease leads to high morbidity and mortality in piglets [18,20]. These observations were in agreement with those found in this case report.

**Table 2.** Presence of PDCoV post-feedback and homogenization in samples of sows that did not present symptoms compatible with PEDV and PDCoV.

N° pool^a^	CT PEDV	CT PDCoV	CT IC	PEDV result	PDCoV result
1	–	19	25	Negativo	Positivo
2	–	25	26	Negativo	Positivo
3	–	20	23	Negativo	Positivo

Signs “–” in the column CT PDV means that there was no reaction.

a Each pool had the capacity of five samples.

**Table 3.** PDCoV verification in negative gilts prior to movement within the pig farm.

Pools^a^ (quantity)	CT PEDV	CT PDCoV	CT IC	PEDV results	PDCoV results
5	–	–	28	Negativo	Negativo
4	–	–	29	Negativo	Negativo
6	–	–	30	Negativo	Negativo
15	–	–	31	Negativo	Negativo
11	–	–	32	Negativo	Negativo
6	–	–	33	Negativo	Negativo
8	–	–	34	Negativo	Negativo
2	–	–	35	Negativo	Negativo
2	–		36	Negativo	Negativo

a Each pool contained five samples (5 × 59 = 295 individual samples).

Given the difficulties in the diagnosis due to clinical evidence, the method used to diagnose PDCoV through qRT-PCR [12] and RT-PCR [9] was efficient. The speed and safety of the technique allowed the timely implementation of the control and eradication program.

In the studies by Chen *et al.* [20] and Jung *et al.* [3], the viral agent caused clinical signs such as vomiting, watery diarrhea, dehydration, and loss of appetite in conventional and gnotobiotic piglets between 24 and 72 h after contact, and they were maintained for 7–10 days. These clinical signs mentioned were the same as those found in the present case. According to Ma *et al.* [21], the cessation of fecal excretion occurs between 27 and 35 days, even up to 42 days, and the surviving pigs begin to produce antibodies from day 7 to 14, after the encounter with the virus. In the present case that is described, the time of presentation of diarrhea in piglets was 7–12 days, and in sows between 24 and 72 h post-inoculation it remained between 5 and 7 days.

The implementation of the feedback and homogenization program in this case report was similar to those used in the control and eradication of PEDV [8,14], but with improvements in the intake procedures of samples, sequential monitoring, and improvement of biosecurity (dry showers and transit changing rooms). In the case of PDCoV in this report, disease control was carried out for 9 weeks (63 days; from feedback to the first negative sampling), and eradication was carried out for 252 days (36 weeks). On the other hand, the time from the feedback (exposure) until there were four consecutive samples of negative to PEDV in the pig farm [14] was 6.5 months (196 days), depending on other diseases present.

An additional study on the characterization of the complete genome on the PDCoV virus in Peru showed that we were dealing with a strain closely related to the North American strains, and given the phylogenetic analysis, the Peruvian strain would have originated from North American strains [16]. To date, it is not known what the means of entry into the country would have been.

Some limitations have been identified in the implementation process of the control and eradication program for this disease. Scarce information on control strategies of the new agent, the wide distance between the diagnostic centers and the pig farm, and the high cost of sample analysis were the most important.

## Conclusion

Rigorous implementation of a feedback and homogenization program, farm closure, measures to improve farm and animal health, and respective monitoring made it possible to control and eradicate PDCoV disease in 252 days (36 weeks), after a rapid and safe diagnosis of the disease using molecular techniques.

## List of Abbreviations

PDCoV = porcine deltacoronavirus; PEDV = porcine epidemic diarrhea virus; GETV = porcine gastroenteritis virus; RT-PCR = real-time polymerase chain reaction; qRT-PCR = quantitative real-time reverse transcription.
